# Characterization of oral virome and microbiome revealed distinctive microbiome disruptions in paediatric patients with hand, foot and mouth disease

**DOI:** 10.1038/s41522-021-00190-y

**Published:** 2021-02-19

**Authors:** Si Xian Ho, Nyo Min, Emmerie Phaik Yen Wong, Chia Yin Chong, Justin Jang Hann Chu

**Affiliations:** 1 Laboratory of Molecular RNA Virology and Antiviral Strategies, Department of Microbiology, Yong Loo Lin School of Medicine, National University of Singapore, Singapore, Singapore; 2 Infectious Disease Translational Research Programme, Yong Loo Lin School of Medicine, National University of Singapore, Singapore, Singapore; 3 Infectious Disease Service, Department of Pediatrics, KK Women’s and Children’s Hospital, Singapore, Singapore; 4 Collaborative and Translation Unit for HFMD, Institute of Molecular and Cell Biology, Agency for Science, Technology and Research (A*STAR), Singapore, Singapore

**Keywords:** Metagenomics, Metagenomics

## Abstract

While the underlying determinants are unclear, hand, foot and mouth disease (HFMD) presents a wide spectrum of clinical manifestations with varying severity in different individuals. Recently, many studies identified the human microbiome as a critical factor in the pathogenesis of various diseases. Therefore, we here investigated the ecological dynamics of the oral microbiome changes during the HFMD infection. After targeted enrichment of all known vertebrate viruses, the virome profiles of symptomatic and asymptomatic HFMD patients were examined and revealed to be significantly altered from those of healthy individuals, with nine discriminative viruses detected. Further characterization of the prokaryotic microbiome revealed an elevated level of *Streptococcus* sp. as the most important signature of the symptomatic HFMD cohort, positively correlating to the level of enterovirus A RNA. In addition, we found that while coxsackievirus A5 is detected in saliva RNA of all asymptomatic cases, coxsackievirus A6 dominates the majority of the symptomatic cohort.

## Introduction

Hand, foot and mouth disease (HFMD) is a highly contagious common viral childhood illness. While HFMD is normally a mild and self-limiting disease, severe complications with the nervous and respiratory system, such as encephalitis, and pulmonary haemorrhage can occur, which often led to death or permanent paralysis^1,2^.

Human enteroviruses, belonging to the genus *Enterovirus* and under the family *Picornaviridae*, are the aetiologic agents of HFMD, with >20 different serotypes described^3^. Initially, human enteroviruses A71 (EV-A71) and coxsackievirus A16 (CV-A16) were regarded as the major serotype responsible for HFMD outbreaks^4–6^. However, in recent years, HFMD caused by CV-A6 has become increasingly common^7^. It was first associated with HFMD in Finland^8^ and Singapore^9^ outbreaks in 2008, and subsequently, CV-A6-associated HFMD outbreaks became widespread in other parts of Europe and Asia^3,10,11^, as well as in North America from 2011 to 2012^12,13^. Since then, CV-A6 has been identified as an emerging predominant agent of HFMD epidemic globally^14^.

However, in certain cases of HFMD, enteroviruses that were associated with HFMD could not be detected from several mild to severe cases of HFMD using routinely used molecular diagnostic assays such as real-time polymerase chain reaction (PCR)^15^. Metagenomics analysis on enterovirus reverse transcription-PCR-negative faecal samples revealed the possibility of co-circulation of multiple members under the genus enterovirus in an HFMD outbreak^15^. Apart from the detection of EV-A71 (0.07%) and CV-A16 (0.43%), which are commonly associated with HFMD infection, HRV-C (45.2%), CV-A21 (34.1%), CV-A10 (24.1%) and nine other enteroviruses (9.02%) were also detected. Co-infection with enteric viruses were also found in these faecal samples, suggesting that a mild virus infection could be aggravated during co-infection through upregulated immune response, leading to increased morbidity risk^15^. Another study has found serotypes of enteroviruses to be different between the severe and mild HFMD patients^16^. Serotypes of enteroviruses in mild cases were found to be more complex and diverse as compared to the severe cases, with 9 out of 12 serotypes that were exclusively identified only in mild HFMD patients^16^. Co-infections were also reported to occur and are more frequent in mild than in severe HFMD patients^16^. In mild cases, co-circulation of diarrhoea-related viruses were more common, while severe cases were more prone to co-circulate with common respiratory viruses^16^.

Given that non-HFMD-associated viruses were also often detected in HFMD patients, there could be a potential relationship between non-enteroviruses in the virome and HFMD-associated enteroviruses that ultimately affecting the HFMD pathogenesis. Moreover, much is still not known about the asymptomatic populations even though the cases could be as high as 71% in the community setting^17^. Through the neglected HFMD asymptomatic population, we may be able to determine if certain groups of viruses are responsible for the asymptomatic phenotype. Virome of healthy individuals will also be investigated to identify if there is a shift in virome profile upon infection with enteroviruses associated with HFMD. As virome is not the sole component of the oral microbiome, trans-kingdom interactions between the virome and prokaryote microbiome will also be explored to relate their patterns to each of the healthy, symptomatic and asymptomatic cohorts for HFMD.

## Results

### Salivary virome composition

**Fig. 1:**
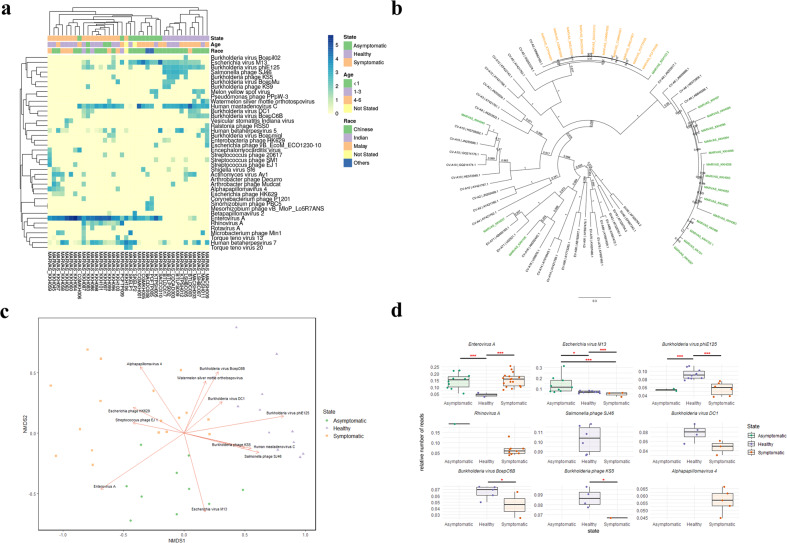
Comparison of salivary virome in healthy, symptomatic and asymptomatic for HFMD samples (*n* = 38). **a** Heatmap of the top 40 viruses with the highest relative number of reads detected in saliva samples. **b** Phylogenetic tree of enterovirus A detected in symptomatic and asymptomatic samples based on the viral protein 1 gene sequences. Symptomatic samples are coloured in green and asymptomatic samples are coloured in orange. Bootstrap values are shown at the branch nodes and the scale bar represents the genetic distance. KKH103 was found to have dual infection of both CV-A5 and CV-A6, labelled as KKH103 1 and KKH103 2. **c** NMDS ordination plot using Bray–Curtis distance metric of saliva virome with vectors representing correlated viruses. Vectors represent viruses that influence each axis, only significant viruses with *p* value <0.05 were shown **d** Boxplots showing the distribution of the relative number of viral reads for each virus detected in the salivary virome. Only the top 40 viruses that were detected with the significance of *p* value <0.05 between at least two of the cohorts, and viruses that were detected exclusively in one cohort were shown. Boxplots display the median distribution (centre line) and boxes show the interquartile range. Whiskers extending out of the boxes mark the minimum and maximum observed values for each dataset. Kruskal–Wallis test followed by Dwass–Steel–Critchlow–Fligner post hoc test were used to generate *p* values and is represented as a red asterisk above the boxplot. (*p value < 0.05, ***p value < 0.001).

Fifty-five children were enrolled in this study between June 2013 to January 2018 with socio-demographic profiles described in Supplementary Tables 1 and 2. The distribution of the saliva samples for virome and prokaryotic microbiome analysis is provided in Supplementary Fig. 1. A total of 171 viruses were detected from healthy (*n* = 11), symptomatic (*n* = 17) and asymptomatic (*n* = 10) for HFMD saliva samples. The relative number of reads for each of the virus species was calculated using the number of reads assigned to the virus divided by the total number of reads generated in the saliva sample. The viruses were then ranked by their relative number of reads and the top 40 viruses were hierarchically clustered in a heatmap (Fig. 1a). Bacteriophages (*n* = 27, 67.5%) accounted for most of the top 40 viruses, followed by vertebrate viruses (*n* = 11, 27.5%) and plant viruses (*n* = 2, 5%). Analyses revealed groups of vertebrate viruses that were ubiquitous across three cohorts; human mastadenovirus C (HAdV-C), the most common cause of respiratory disease in young children, was detected in 71% (27/38) of the samples. Viruses belonging to the *Herpesviridae* family were also widespread among the saliva samples, with 71% (27/38) of the samples harbouring human betaherpesvirus 7. Another member of the *Herpesviridae* family, human betaherpesvirus 5 (18/38), was also found, albeit at a lower percentage (47.3%). While HAdV-C and herpesviruses are known to highly repetitive regions in the genome, which could potentially lead to false identification, BLAST search with default parameters showed specific matches to HAdV-C (Supplementary Table 4), human herpesviruses 5 and 7, respectively (Supplementary Table 5). Other reads that were identified to be human betaherpesvirus 6b and human gammaherpesvirus 4 were regarded as contaminants from the human genome due to high sequence identity to endogenous herpesvirus 6b and human genome chromosome 6, respectively (Supplementary Table 6).

The aetiological agent of HFMD, enterovirus A, was detected in all of the saliva samples, obtained from patients symptomatic for HFMD and also from asymptomatic children. Asymptomatic individuals were defined wherein subjects do not exhibit any HFMD-associated symptom but were pan-entero PCR positive at the point of sample collection. Interestingly, the level of enterovirus in the symptomatic group was higher, with an average read count of 7,051,937, as compared to the asymptomatic cohort, with an average read of 2454. Further genotyping of the enterovirus A detected in the two cohorts using phylogenetic methods in Geneious software (Biomatters Ltd, Auckland, New Zealand), identified CV-A6 to be the dominant genotype (77.7%) of the symptomatic cohort (Fig. 1b). Other genotypes detected were CV-A16 (5.5%), EV-A71(5.5%) and CV-A10 (5.5%). Conversely, enterovirus A in the asymptomatic samples was revealed to be CV-A5. One of the symptomatic samples were found to have a dual infection of both CV-A6 and CV-A5.

### Distinct virome profile among healthy, symptomatic and asymptomatic for HFMD cohort

To investigate if other members of the virome could discriminate each of the healthy, symptomatic and asymptomatic for HFMD cohort, non-metric dimensional scaling (NMDS) ordination plot using Bray–Curtis distances was constructed to visualize the differences in viral community between the three cohorts. NMDS plot was able to discriminate the virome profiles of the three cohorts, with minimum overlaps (Fig. 1c). Analysis of similarities (ANOSIM) was subsequently performed to investigate the level and significance of separation between the three cohorts. ANOSIM indicated separation between the three cohorts with significance (*R* = 0.5271, *p* value = 0.0001). Influences of potential confounding variables on clustering of samples were further evaluated in NMDS and no clear clustering based on age and race can be observed (Supplementary Fig. 4a, b). Expectedly, enterovirus A was the determining driver for distinct clustering of healthy cohorts from the symptomatic and asymptomatic cohort (*p* value <0.001). In addition, *Salmonella* phage SJ46 along with *Burkholderia* virus phiE125 were the major determinants for the discrimination between the two groups (*p* value <0.001). *Burkholderia* virus phiE125 were shown to have a significantly higher relative number of reads in healthy control as compared to the symptomatic and asymptomatic for HFMD cohort, while *Salmonella* phage SJ46 were detected exclusively in healthy controls (Fig. 1d). Asymptomatic samples tend to cluster at the bottom of the NMDS plot, which was largely influenced by *Escherichia* virus M13. Conversely, symptomatic samples tend to cluster at the top left of the NMDS plot, which was influenced by *Escherichia* phage HK629, *Streptococcus* phage EJ1 and Alphapapillomarvirus 4. Taken together, these findings suggest an association of certain group of vertebrate viruses and bacteriophages with the clinical outcome of HFMD apart from the aetiological agent.

### Salivary bacterial composition

**Fig. 2:**
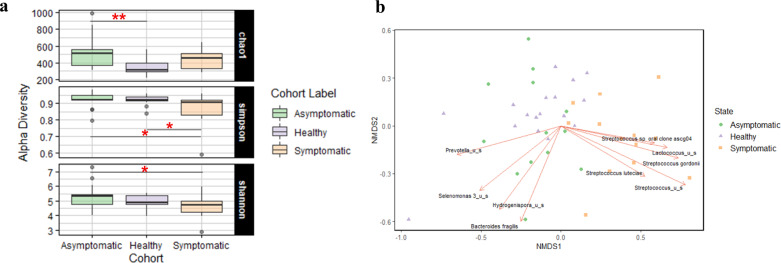
Comparison of salivary prokaryotic microbiome in healthy (*n* = 17), symptomatic (*n* = 13) and asymptomatic HFMD samples (*n* = 13). **a** Alpha-diversity analysis using Chao1 index, Shannon–Wiener and Simpson index analysis. Boxplots display the median distribution (centre line) and boxes show the interquartile range. Whiskers extending out of the boxes mark the minimum and maximum observed values for each dataset. Kruskal–Wallis test followed by Dwass–Steel–Critchlow–Fligner post hoc test were used to generate *p* values and is represented as a red asterisk above the boxplot. (*p value < 0.05, **p value < 0.01) **b** NMDS ordination plot using Bray–Curtis distance metric of saliva prokaryotic microbiome with vectors representing correlated bacteria. Vectors represent bacteria that influence each axis, only significant bacteria with *p* value < 0.01 were shown.

As virome profile was distinct for each of the cohort, we would like to know if the bacterial community was also altered. As such, 16S ribosomal RNA (rRNA) gene sequencing was conducted on the saliva samples. Proteobacteria, Firmicutes, Bacteriodetes and Actinobacteria were the four most abundant phyla that predominated in all of the cohorts. The most commonly detected genera with the highest relative number of read counts in all populations was *Streptococcus*, with at least 13 different species detected. Alpha diversity across the asymptomatic, symptomatic and healthy cohorts were computed using Chao1, Shannon and Simpson indices (Fig. 2a). The analysis revealed symptomatic cohort to have significantly lower Shannon (*p* value = 0.043) and Simpson diversity (*p* value = 0.048) index as compared to the asymptomatic cohort, with samples in the symptomatic cohort exhibiting a lower alpha diversity. Significant increase in microbial richness in the asymptomatic cohort was observed compared to the healthy cohort (*p* value = 0.004). Using NMDS with Bray–Curtis distance matrix, bacterial composition data of all three cohorts reveals slight separation between the three cohorts. Influences of potential confounding variables on the clustering of samples were further evaluated in NMDS and no clear clustering can be observed (Supplementary Fig. 4c, d). Clustering of symptomatic cohort away from the healthy and asymptomatic cohort is associated with the presence of *Streptococcus* spp. and *Lactococcus* sp. in the symptomatic samples (Fig. 2b). However, there is no clear distinction between the asymptomatic and healthy clusters based on the bacteriome profile.

### Alteration of the oral prokaryotic microbiome

To further understand the differences in the prokaryote profile, Kruskal–Wallis test followed by the Dwass–Steel–Critchlow–Fligner comparison test was conducted with each of the prokaryotes of the prokaryotic microbiome. A total of 120 bacteria was found to be significantly different in a relative number of read counts between at least two of the cohorts (Supplementary Fig. 2). Of the 120 bacteria, 46.6% were defined as normal oral flora by previous studies^18,19^. Fifty percent (28/56) of the normal oral flora were found to be significantly lowered in the symptomatic population as compared to the healthy controls, but remains elevated in the asymptomatic population. On the other hand, 14.3% (8/56) of the normal oral flora were significantly elevated in the symptomatic population. Notably, *Streptococcus* spp., which were identified as the driver to clustering of symptomatic samples in the NMDS plot, were elevated in the symptomatic population. A small percentage of the normal flora (10.7%) were significantly depleted in both symptomatic and asymptomatic population. Among the remaining bacteria groups that were not defined as part of the normal oral flora, 34.4% (22/64) were depleted in the symptomatic population as compared to the healthy controls. A higher proportion (26.6%) of this group of bacteria, as compared to the normal flora, were elevated in the symptomatic population. In all, 15.6% (10/64) of bacteria that were not defined as part of normal oral flora were elevated in the asymptomatic population.

**Fig. 3:**
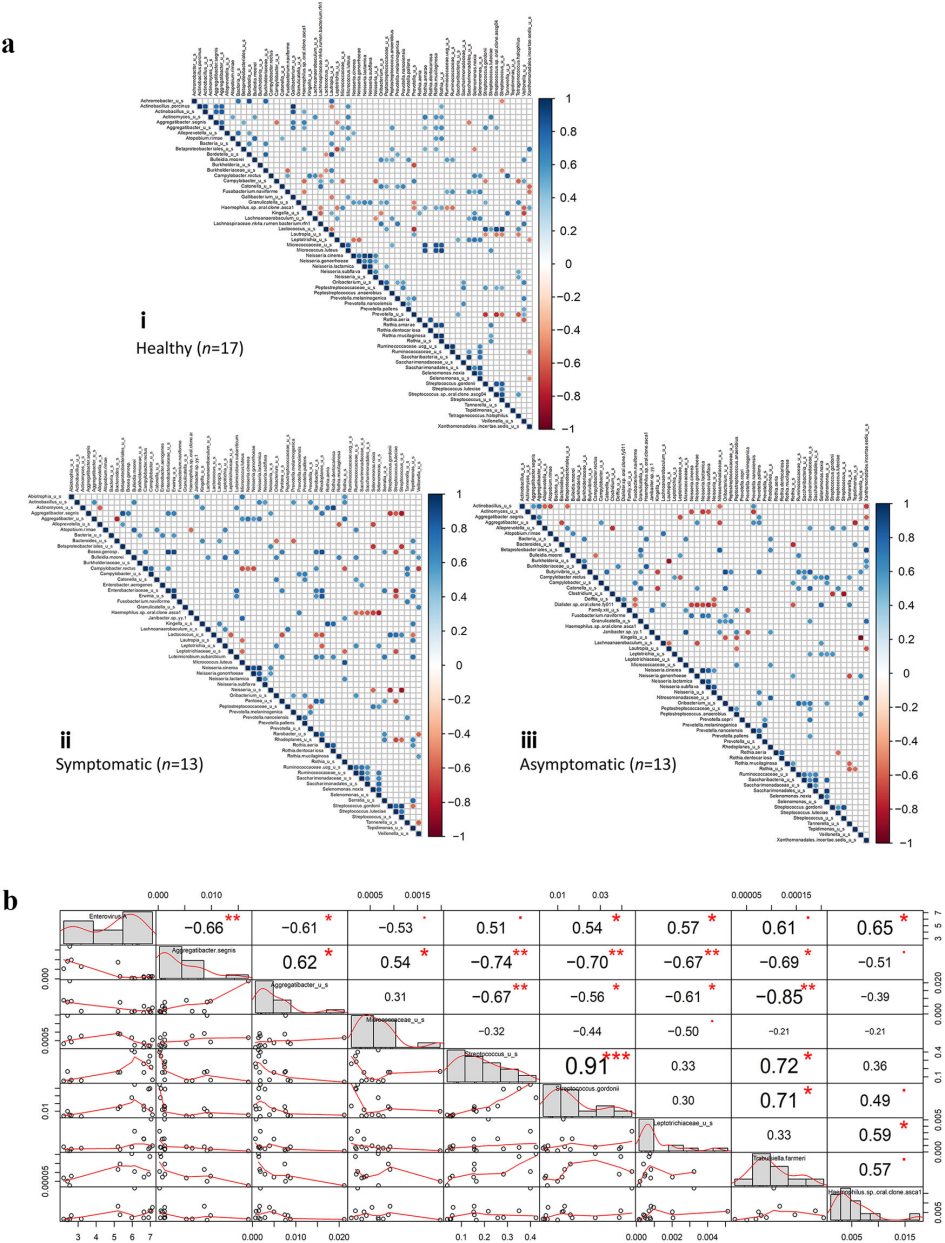
Correlation analysis of prokaryotic microbiome. **a** Pairwise correlation matrix among all bacterial species in (i) healthy, (ii) symptomatic and (iii) asymptomatic populations. Spearman’s correlation was generated using *corrplot* R package. Only significant correlation with *p* value < 0.05 were shown. **b** Correlation analysis between enterovirus A and bacteria in both symptomatic (*n* = 11) and asymptomatic (*n* = 6) cohort. Only bacteria with at least Spearman’s correlation >0.4 with enterovirus A were included. The distribution of each sample is represented as a histogram. Bivariate scatter plots of a relative number of reads with a fitted line are displayed with each point representing an enterovirus-infected sample (both asymptomatic and symptomatic). The value of the correlation between the two members of the microbiome was indicated with significance level in red symbols. Significant correlations were marked with either red asterisk or red dot (**p value ≤ 0.001; *p value ≤ 0.01; ^•^*p* value ≤ 0.05).

Interbacterial correlations between the members of the prokaryotic microbiome were also explored using spearman correlation to investigate if the microbiome spectrum was altered. A shift in correlation profile within the prokaryote community in the microbiome can be observed when comparing the healthy, symptomatic and asymptomatic cohort (Fig. 3a and Supplementary Fig. 3). For instance, *Haemophilus* sp. oral clone asca1 was positively correlated to five other bacteria and negatively correlated to six other bacteria in the healthy population. However, in the symptomatic population, *Haemophilus* sp. oral clone asca1 was negatively correlated to another group of bacteria, mainly members of the Firmicutes, which was not observed in the healthy controls. In the asymptomatic population, no significant correlation with other members of the prokaryotic microbiome was observed for *Haemophilus* sp. oral clone asca1.

### Enteroviruses and prokaryotic microbiome interactions

To investigate if changes in the prokaryotic microbiome were driven by enteroviruses, the correlation between enteroviruses and bacteria was determined. Correlation analysis revealed eight different bacteria species to be significantly correlated with the relative number of reads of enterovirus A in symptomatic and asymptomatic samples (Fig. 3b). *Aggregatibacer segnis*, *Aggregatibacter* sp. and *Micrococcaceae* sp. were negatively correlated to enterovirus A, while *Streptococcus* sp., *Streptococcus gordonii*, *Lepotrichiaceae* sp., *Trabusiella farmeri* and *Haemophilus* sp. oral clone asca1 were positively correlated to enterovirus A. Enterovirus A was most positively correlated to *Haemophilus* sp. oral clone asca1 (*r* = 0.65), while most negatively correlated to *A. segnis* (*r* = −0.66). Significant correlation within the prokaryotic community itself can also be observed, with members under the genus of *Streptococcus* being highly positively correlated (*r* = 0.91).

## Discussion

HFMD is a disease that can have multiple clinical manifestations. Apart from the typical clinical characteristics of blister-like rashes on the hands, feet and buttocks, as well as ulcers in the mouth, atypical manifestations of HFMD were also described^20^. This includes neurological manifestations that can be fatal^2^. On the other hand, infection with enteroviruses can be asymptomatic; however, the true incidence of asymptomatic infection remains unknown^21^. Currently, there is no antiviral treatment available for HFMD, with the main management of the disease being symptomatic relief. Therefore, elucidating the virome and prokaryotic microbiome of HFMD may offer new insights into the disease pathogenesis and providing novel prevention and treatment strategies. Here, we made several findings that are of potential medical significance.

In this study, majority of the symptomatic samples were identified to be CV-A6, suggesting that CV-A6 remains as the predominant circulating virus serotype in Singapore. This is supported by a surveillance study on the communicable disease in Singapore reported by Ministry of Health in 2017 where coxsackieviruses type A (22.0%) were identified to be the majority serotype, followed by EV-A71 (3.7%)^22^. Among all the coxsackieviruses type A, CV-A6 was the predominant serotype, accounting for 54.0% of the cases^22^. Conversely, all of the enterovirus A detected in the asymptomatic cohort of this study were CV-A5. This suggests a possible association of CV-A5 serotype to the asymptomatic phenotype in Singapore, along with the fact that it was present in a lower amount. While CV-A5 was rarely the cause of HFMD outbreak, it has been sporadically isolated from HFMD patients elsewhere^6,23^. In one of the studies in China, CV-A5 were detected in patients with severe, non-fatal HFMD^6^. However, the extent of CV-A5 infection and their implication on HFMD in Singapore is unknown which warrants further surveillance on CV-A5 serotype.

Over the past decade, the epidemiology of HFMD has changed substantially^24^. Co-circulation of multiple serotypes in the community was often observed, which could potentially increase the recombination events, sprouting new causative agents that may eventually affect the virulence of viruses during outbreaks^6^. Therefore, it is essential to not only focus on the predominant causative serotype but also on this group of the asymptomatic population, which were often neglected in epidemiology studies.

Most prior studies on oral or salivary virome have established that it is dominated largely by phage communities, with few eukaryotic viruses such as torque teno viruses, circoviruses, herpesviruses and Epstein–Barr virus being detected^25–27^. These studies relied on conventional methods for viral enrichment, such as filtration, nuclease digestion or cesium chloride density gradient centrifugation, but none have specifically explored the small community of vertebrate viruses. In this study, vertebrate viruses were especially enriched using VirCapSeq-Vert probes that were designed to target all known vertebrate viruses; however, the majority of the viruses detected were still bacteriophages, suggesting that indeed saliva do not harbour much vertebrate viruses. Similar to previous studies on oral virome, herpesviruses were one of the most widespread eukaryotic viruses that were detected in saliva^27^. However, the virus that was detected to be the most widespread was HAdV-C, which was present in 71% of the samples. HAdV-C is the most common cause of respiratory disease in young children and can persist as asymptomatic carriers until at least their young adulthood^28^. To our best knowledge, this is the first time that HAdV-C has been described as a possibility of being a commensal virus in the saliva of children.

One inherent limitation of the study is that as we needed to detect both RNA and DNA mammalian viruses in a cost-effective manner, DNA and RNA were sequenced together, which in turn renders detected transcriptional RNA indistinguishable from genomic RNA or DNA. This can be addressed in future studies by performing RNA or DNA digestion before sequencing, which would allow the segregation of transcripts from genomic materials for DNA viruses. However, as nucleases would digest both genomic and transcriptomic RNA indiscriminately, such an approach would not work for transcript detection of RNA viruses, including the enteroviruses that we detected in many HFMD samples.

The distinct clustering of samples based on hierarchical clustering in the heatmap suggests that virome profile were different in the three cohorts that were further illustrated from the NMDS plot in which the virome profile of healthy samples was distinctly different from the symptomatic and asymptomatic samples. Apart from enteroviruses, bacteriophages were demonstrated to have discriminating power between different cohorts. Elevated levels of *Salmonella* phage SJ46 and *Burkholderia* virus phiE125 in the healthy controls as compared to the other two cohorts suggests an association to the healthy state. *Escherichia* virus M13 was able to discriminate asymptomatic from symptomatic samples, with a higher relative number of reads detected in the asymptomatic samples. However, as this study specifically enriched for vertebrate viruses, the diversity of bacteriophages may be underestimated. This can be further explored using bacteriophage-specific screen. Nevertheless, this study still demonstrated that bacteriophages population varied in a relative number of reads in the healthy, symptomatic and asymptomatic cohort and was associated with the healthy state. This suggests the possibility of bacteriophages as a potential player in modulating HFMD.

The shift in microbiome from the healthy state was observed in the symptomatic and asymptomatic HFMD population, in which 44.6% of the shifted microbiome were part of the normal oral flora as defined by other studies. Normal flora was found to be present in good amount in the healthy controls, but some were significantly depleted in the symptomatic population and yet remained elevated in the asymptomatic population. While enterovirus is present in both symptomatic and asymptomatic samples, the degree to which the prokaryotic community was impacted differs. This suggests that a different immune response may be occurring between the symptomatic and asymptomatic population such that in the asymptomatic population, the immune response was insufficient to modulate this group of bacteria. A large proportion of bacteria that were not defined as normal oral flora were elevated in the symptomatic population, suggesting that depletion in normal flora in the symptomatic state may serve as an opportunity for this group of transient bacteria to thrive.

The most important prokaryotic microbiome signature of the symptomatic cohort was an elevated level of *Streptococcus* spp., which distinguished the symptomatic cohort from the healthy and asymptomatic cohort as seen in the NMDS plot. *Streptococci* are universally present in all oral sites and are the dominant genus in saliva^29,30^. Unsurprisingly, the most commonly detected bacteria in this study was *Streptococcus* spp. They are the first colonizer of the oral cavity and is known to play an important role in shaping the oral microbiota due to high-affinity adhesins that can mediate binding^31^. Therefore, an elevation in *Streptococcus* spp. may significantly affect other prokaryotic members, causing alteration from the healthy state.

Our study suggests that changes in prokaryotic microbiome could be driven by enterovirus, as enterovirus were shown to be correlated to eight other bacteria. Strong correlations within bacteria community itself were also observed. Viral–bacterial interactions have been demonstrated to be a major contributor to multiple diseases in which the viruses were able to promote bacterial colonization, while bacteria could also promote viral infection^32,33^. Therefore, it is likely that in this case of HFMD, enterovirus could directly modulate this group of driver bacteria and as such altered the overall prokaryotic ecology and affects how bacteria groups interact with one another. *Streptococcus* sp. positive correlation with enterovirus A in this study is definitely interesting and warrants further research to probe into studies of possible trans-kingdom interactions that can lead to clinically relevant druggable targets.

This study has successfully mapped out both the virome and prokaryotic microbiome of the salivary virome in healthy, symptomatic and asymptomatic for HFMD cohort, showing that microbiome profile was altered from the healthy state during enterovirus infection. This suggests that the manifestation of HFMD may occur in a combinatorial manner with other members of the microbiome instead of solely based on the aetiological agent enterovirus itself. Enteroviruses were shown to be associated with a disruption in the prokaryotic microbiome and is likely through a group of driver bacteria that were identified to be significantly correlated to enterovirus. In addition, different serotypes were identified for symptomatic and asymptomatic population, which also contributes to the differing phenotype observed. This study has increased the understanding of the microbiome spectral of HFMD thus highlights that HFMD may be influenced by other members of the microbiome.

## Methods

### Clinical samples

Saliva samples were collected from KK Women’s and Children’s Hospital and approved childcare centres in compliance with a protocol with prior approval from Singhealth centralized institutional review board under CIRB number 2018/3181 and NUS-IRB (reference no. B-14 273). All participants have provided written consent to participate in this study. The inclusion criteria for symptomatic HFMD cohort is as such: patients were diagnosed HFMD positive by paediatricians according to World Health Organization guidelines between June 2013 to January 2018 and the in-house pan-entero PCR must be tested positive. The healthy cohort samples were collected routinely from childcare centres and the inclusion criteria were as such: the participant must not display HFMD-associated symptoms such as fever, rashes on palm and hand and soles of feet, as well as an ulcer in the mouth and blisters at the point of sample collection, and 4 weeks prior, the pan-entero PCR must also be tested negative. If a subject whose sample is routinely collected from a childcare centre, who did not display any symptom of HFMD as previously described, tested positive for pan-entero PCR, the participant is then included in the asymptomatic cohort. All saliva samples were collected using SalivaBio Children’s swab (Salimetric Inc., Carlsbad, CA). Subjects were asked to refrain from eating and drinking 3 h prior to collection of saliva. Rinsing of the mouth was not done to prevent dilution of the sample. Saliva was collected by placing one end of the swab under the subject’s tongue securely for 2 min. The swab was folded and placed in the SalivaBio collection tube and stored in dry ice immediately after collection. Swabs were centrifuged at 1500 × *g* for 1 min before transferring supernatant to nuclease-free tubes and stored at −80 °C until used.

Pan-enterovirus PCR was conducted as previously described^34^. Amplicons were visualized under ultraviolet light after gel electrophoresis and stained with ethidium bromide. An expected 154 bp PCR product is an indication of pan-enterovirus positive.

The viral mock community was generated by combining purified preparations of laboratory stocks coxsackievirus A6, coxsackievirus A16, chikungunya, dengue virus 2 and Zika virus. The viral mock community was used for assessment of background contamination from experimental reagents and procedures.

### Virome sequencing and data analysis

Total RNA was extracted from the saliva samples using acid phenol–chloroform (Ambion; 5:1, pH 4.5) extraction with no DNA depletion step. One hundred microlitres of saliva sample was lysed with 3 volumes of lysis/binding buffer (Invitrogen) and mixed thoroughly by vortexing and incubated in ice for 10 min. An equal volume of acid phenol–chloroform 5:1, pH 4.5 (Ambion) was added to the mixture and vortexed for 10 s before centrifuging for 5 min at 21,130 × *g* at 4 °C. The nucleic acid in the aqueous phase was then precipitated with one volume of 100% isopropanol and 2 μl of Pellet Paint® Co-Precipitant (MilliporeSigma). Precipitated RNA was pelleted by centrifugation for 5 min at 21,130 × *g* at room temperature. The pellet was washed three times with 70% ethanol and subsequently three times with 100% ethanol and resuspended in 15 μl of 5 mM Tris-HCl (Sigma-Aldrich).

Extracted RNA was fragmented and reverse transcribed to complementary DNA (cDNA) using Maxima H Minus Double-Stranded cDNA synthesis in accordance with the manufacturer’s protocol (Thermo Fisher Scientific, Massachusetts, USA). The synthesized cDNA was column purified using Nucleospin Gel and PCR Clean-up Kit (Macherey-Nagel, Düren, Germany). Library preparation was performed by following the SeqCap EZ HyperCap Workflow using KAPA Hyper Prep Kit and Single-Indexed SeqCap Adapter Kit A (Roche Diagnostics, Mannheim, Germany). Libraries were quantified using Qubit 2.0 (Life Technologies, Carlsbad, CA, USA) and quality was assessed using Agilent Bioanalyzer DNA 1000 assay (Agilent Technologies, Santa Clara, CA, USA). For enrichment of viral sequences, each pooled library comprising of 11 sample libraries of equimolar was captured using VirCapSeq-VERT capture panel (Roche Diagnostics, Mannheim, Germany)^35^. Captured multiplex libraries were amplified for a total of 14 cycles as recommended by the manufacturer’s protocol and purified with AMPure XP beads before subjecting pooled library to 150 pair-end sequencing on Illumina HiSeq4000. A total of four lanes were used for sequencing of 38 sample libraries, generating an average of 42,578,016 reads per sample.

For virome analyses, pair-end FASTQ files generated from the sequencing were analysed using a cloud-based online classification tool Genome Detective with default parameters (https://www.genomedetective.com/)^36^. Briefly, raw next-generation sequencing reads were adapters trimmed and filtered by removing low-quality reads, human reads and bacterial reads. Remaining candidate viral reads will be sorted, aligned and identified using both nucleotide and protein scores for more sensitive and accurate alignments. To ensure the reliability of our metagenomic results, sequences obtained from the mock community that were deemed as a reagent and environmental contamination will be removed before further analysis (Supplementary Table 3).

### 16S rRNA sequencing and microbiome data analysis

Bacterial genomic DNA was extracted from saliva samples using phenol–chloroform (Sigma; 25:24:1, pH 8.0) method. Extracted DNA samples were subjected to 16S rRNA sequencing. The V3–V4 regions of 16S rRNA were amplified from extracted DNA with universal primers 341F (5′-CCTAYGGGRBGCASCAG-3′) and 806R (5′-GGACTACNNGGGTATCTAAT-3′) primers, using Phusion® High-Fidelity PCR Master Mix (New England Biolabs, Beverly, USA). Amplicons were mixed with an equal volume of 1× loading buffer and visualized at 2% agarose gel. Samples with bright bands between 400 to 450 bp were purified with Qiagen Gel Extraction Kit (Qiagen, California, USA) following the manufacturer’s protocol. Libraries were generated using NEBNext® UltraTM DNA Library Prep Kit and quantified using Qubit and quantitative PCR. Qualified libraries were subjected to sequencing on NovaSeq6000 to generate 250 bp paired-end reads, generating an average of 146,978 reads per sample. After excluding reads shorter than 200 bp and low-quality reads, remaining candidate sequences were clustered with k-mer-based algorithms, 5VCE and NmerCE, and GeneBook reference libraries using CosmosID proprietary platform with default parameters^37^(CosmosID Inc., Rockville, MD).

### Statistical and comparative analysis

Statistical analyses were performed in R version 1.2.1335 using the packages *Hmisc*^38^, *ggplot2*^39^, *corrplot*^40^, performance analytics^41^, *pheatmap*^42^ and *vegan*^43^. Boxplot for relative read counts detected was constructed using *ggplot2*. Spearman’s correlations were performed using *rcorr* function from the *Hmisc* package. NMDS ordinations based on Bray–Curtis distances was performed using *metaMDS* function from R package *vegan* and plot was constructed using *ggplot2*. ANOSIM permutation tests were also performed using *anosim* function in R package *vegan*. Heatmap for visualization of the global virome profile was plotted using online platform ClustVis that uses *pheatmap* package in R.

Alpha diversity of samples for bacteria were analysed by calculating Shannon diversity index, Chao1 and Simpson index, which was estimated using CosmosID pipeline. Plot for alpha diversity was generated using *ggplot2*. Statistical significance between groups was performed using nonparametric analysis of variance Kruskal–Wallis followed by the Dwass–Steel–Critchlow–Fligner comparison test with Jamovi software package version 0.9.6.9.

### Reporting summary

Further information on research design is available in the Nature Research Reporting Summary linked to this article.

## Supplementary information

Supplementary Material

Reporting Summary

## Data Availability

Raw sequences in this study have been deposited in NCBI SRA repository under the BioProject accession no. PRJNA663926.

## Code availability

R script used for analysis is available through figshare at 10.6084/m9.figshare.12137220.
