# Supplementary material for: Characterization of oral virome and microbiome revealed distinctive microbiome disruptions in paediatric patients with hand, foot and mouth disease
Source: NPJ Biofilms Microbiomes. 2021 Feb 19;7:19. doi: 10.1038/s41522-021-00190-y (PMC7895916; doi:10.1038/s41522-021-00190-y)
Supplement: Supplementary file 1 — Supplementary Material [file 41522_2021_190_MOESM1_ESM.pdf]

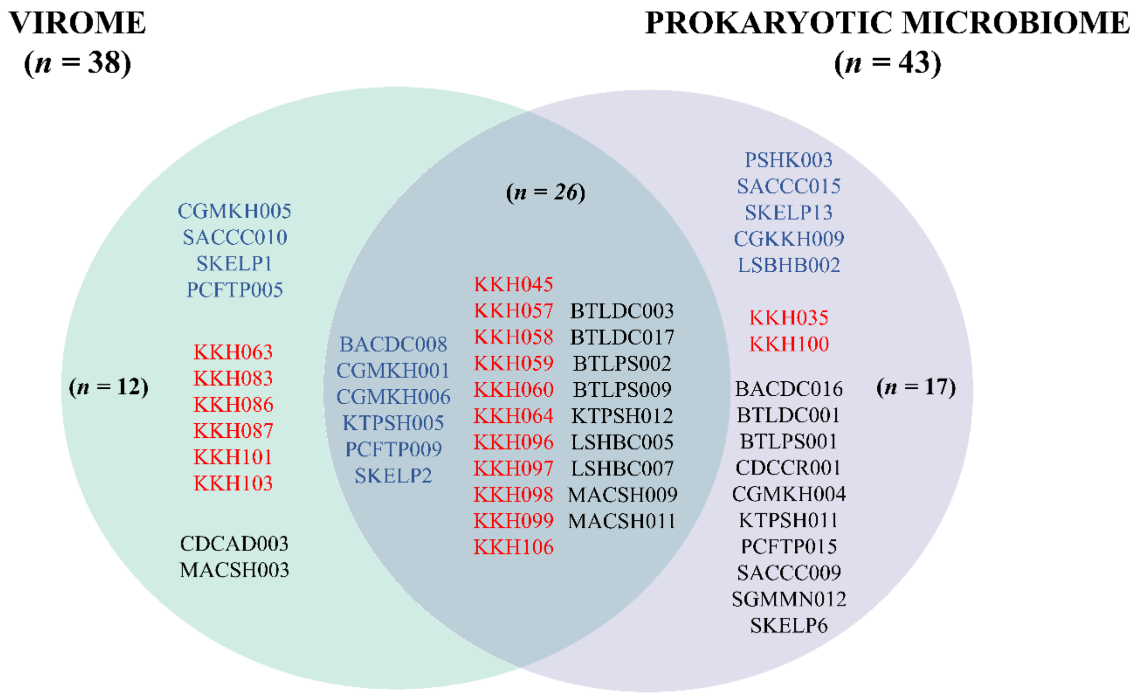

Supplementary Figure 1. Venn diagram showing the list of saliva samples for virome and prokaryotic microbiome analysis. A total of 55 saliva samples were analysed in this study. Of the 55 saliva samples, 12 were analyzed for virome only, 17 were analyzed for prokaryotic microbiome only and 26 were analyzed for both virome and prokaryotic microbiome. Symptomatic samples are coloured in red, asymptomatic sample are coloured in blue and healthy samples are coloured in black.

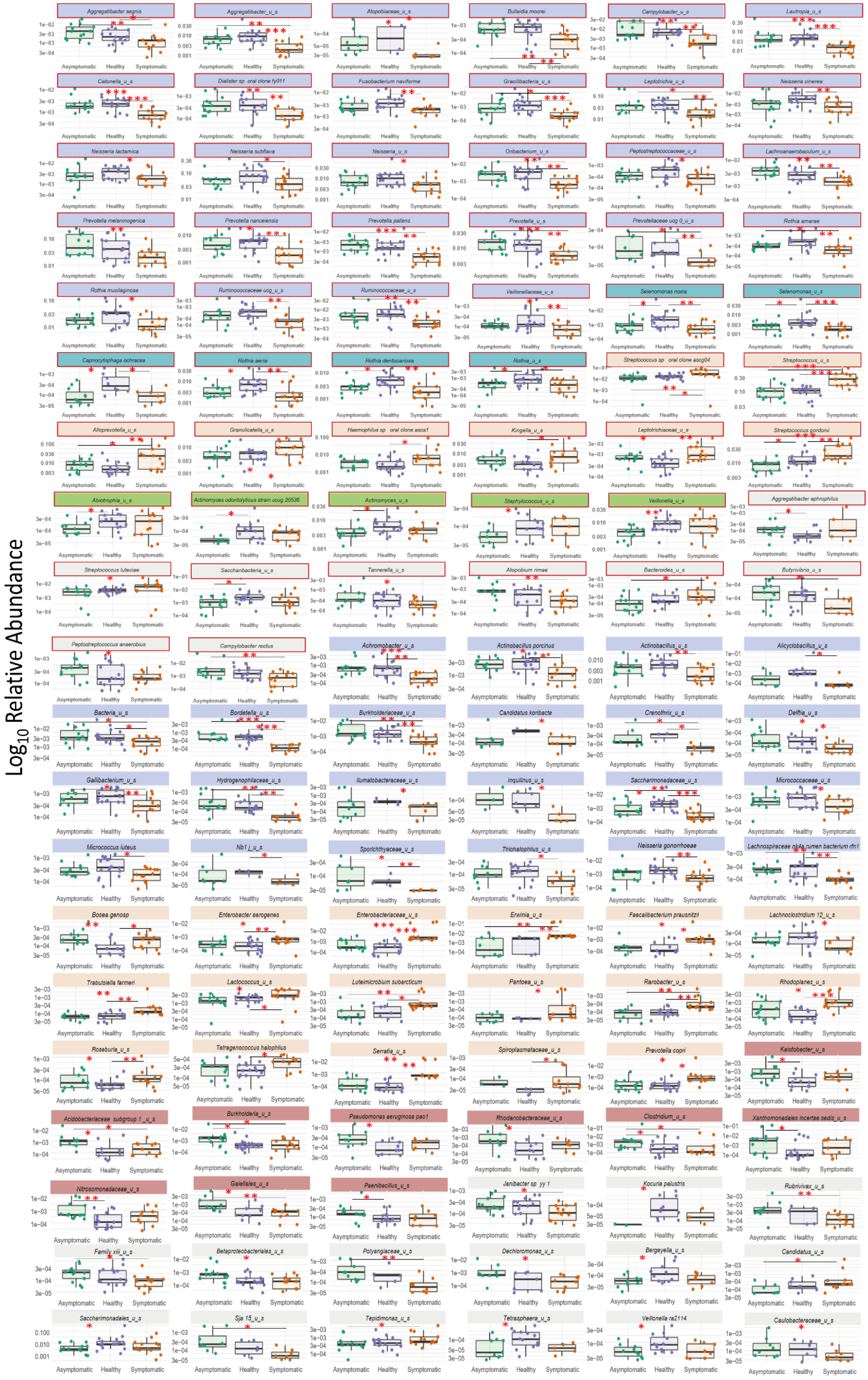
Log10 Relative Abundance
Asymptomatic
Healthy
Symptomatic
Aggregatibacter segnis
Aggregatibacter_u_s
Atopobiaceae_u_s
Bulleidia moorei
Campylobacter_u_s
Lautropia_u_s
Catonella_u_s
Dialister sp oral clone fy011
Fusobacterium naviforme
Gracilibacteria_u_s
Leptotrichia_u_s
Neisseria cinerea
Neisseria lactamica
Neisseria subflava
Neisseria_u_s
Oribacterium_u_s
Peptostreptococcaceae_u_s
Lachnoanaerobaculum_u_s
Prevotella melaninogenica
Prevotella nanceiensis
Prevotella pallens
Prevotella_u_s
Prevotellaceae ucg 0_u_s
Rothia amarae
Rothia mucilaginosa
Ruminococcaceae ucg_u_s
Ruminococcaceae_u_s
Veillonellaceae_u_s
Selenomonas noxia
Selenomonas_u_s
Capnocytophaga ochracea
Rothia aeria
Rothia dentocariosa
Rothia_u_s
Streptococcus sp oral clone ascg04
Streptococcus_u_s
Alloprevotella_u_s
Granulicatella_u_s
Haemophilus sp oral clone asca1
Kingella_u_s
Leptotrichiaceae_u_s
Streptococcus gordonii
Abiotrophia_u_s
Actinomyces odontolyticus strain ccug 20536
Actinomyces_u_s
Staphylococcus_u_s
Veillonella_u_s
Aggregatibacter aphrophilus
Streptococcus luteciae
Saccharibacteria_u_s
Tannerella_u_s
Atopobium rimae
Bacteroides_u_s
Butyrivibrio_u_s
Peptostreptococcus anaerobius
Campylobacter rectus
Achromobacter_u_s
Actinobacillus porcinus
Actinobacillus_u_s
Alicyclobacillus_u_s
Bacteria_u_s
Bordetella_u_s
Burkholderiaceae_u_s
Candidatus koribacter
Crenothrix_u_s
Delftia_u_s
Gallibacterium_u_s
Hydrogenophilaceae_u_s
Ilumatobacteraceae_u_s
Inquilinus_u_s
Saccharimonadaceae_u_s
Micrococcaceae_u_s
Micrococcus luteus
Nb1 j_u_s
Sporichthyaceae_u_s
Thiohalophilus_u_s
Neisseria gonorrhoeae
Bosea genosp
Enterobacter aerogenes
Enterobacteriaceae_u_s
Erwinia_u_s
Faecalibacterium prausnitzii
Lachnoclostridium 12_u_s
Trabulsiella farmeri
Lactococcus_u_s
Luteimicrobium subarcticum
Pantoea_u_s
Rarobacter_u_s
Rhodoplanes_u_s
Roseburia_u_s
Tetragenococcus halophilus
Serratia_u_s
Spiroplasmataceae_u_s
Prevotella copri
Kaistobacter_u_s
Acidobacteriaceae subgroup 1_u_s
Burkholderia_u_s
Pseudomonas aeruginosa pao1
Rhodanobacteraceae_u_s
Clostridium_u_s
Xanthomonadales incertae sedis_u_s
Nitrosomonadaceae_u_s
Paenibacillus_u_s
Janibacter sp yy 1
Kocuria palustris
Rubrivivax_u_s
Family xiii_u_s
Betaproteobacteriales_u_s
Polyangiaceae_u_s
Dechloromonas_u_s
Bergeyella_u_s
Candidatus_u_s
Saccharimonadales_u_s
Sja 15_u_s
Tepidimonas_u_s
Tetrasphaera_u_s
Veillonella ra2114
Caulobacteraceae_u_s

Supplementary Figure 2. Box plots showing relative abundance of bacteria detected in healthy, symptomatic and asymptomatic saliva samples. Box plots were generated using *ggplot2* in R. Only bacteria with statistical significance of p-value <0.05 between at least 2 of the cohorts were shown. Kruskal-Wallis test followed by Dwass-Steel-Critchlow-Fligner post hoc test were used to generate *p*-values. Red border around bacteria name indicates that they are part of normal oral flora defined in other studies. Box plots are colour-coded based on the significant difference between the three cohorts. Purple indicates a significantly higher relative abundance in healthy cohort as compared symptomatic cohort; turquoise indicates significantly higher relative abundance in healthy cohort compared to both asymptomatic and symptomatic cohort; orange indicates significantly higher relative abundance in symptomatic cohort compared to healthy cohort; green indicates significantly higher in healthy compared to symptomatic cohort; grey indicates bacteria that do not fall in any of the above mentioned categories.

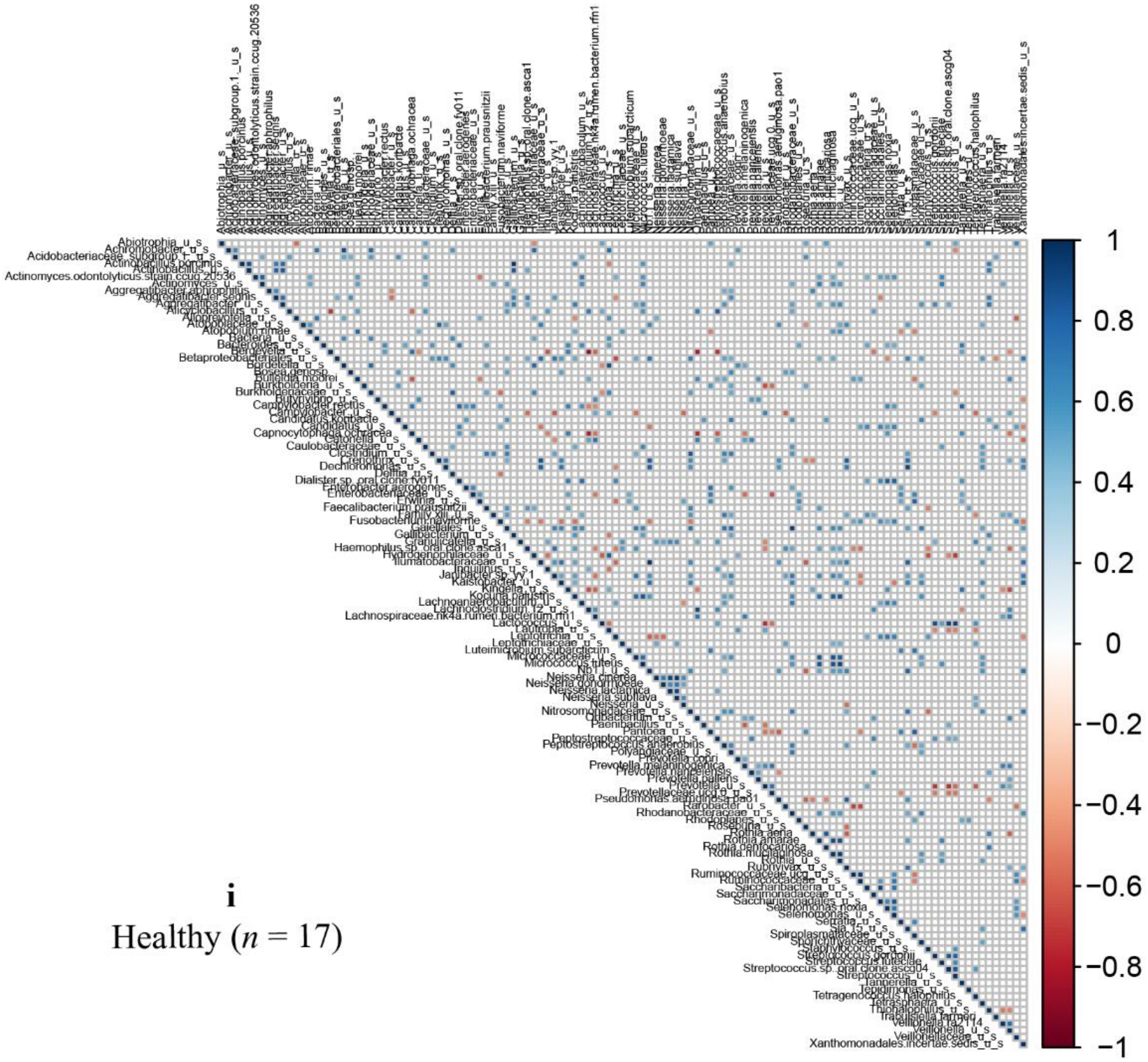

i
Healthy (n = 17)

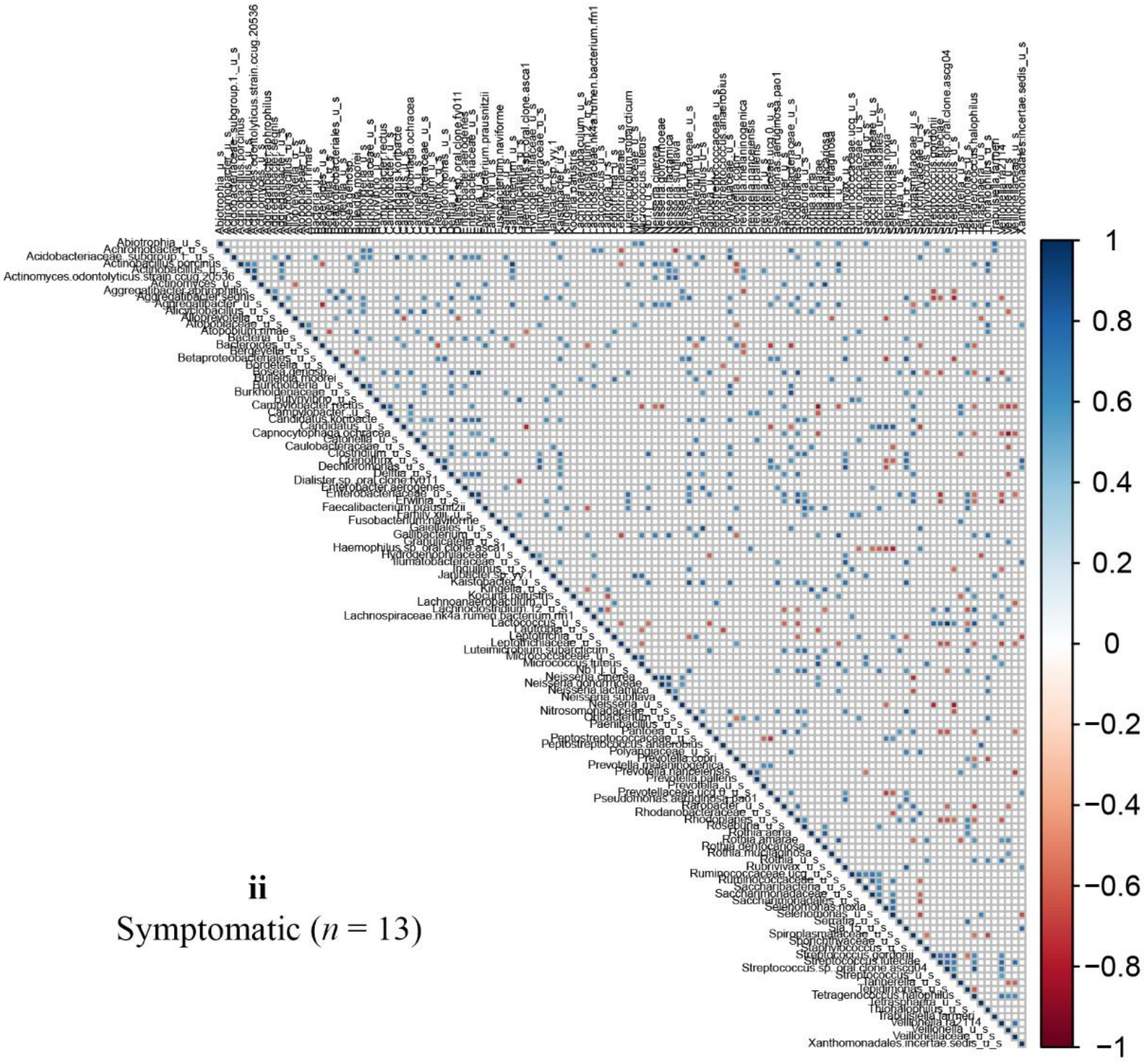

ii
Symptomatic (n = 13)

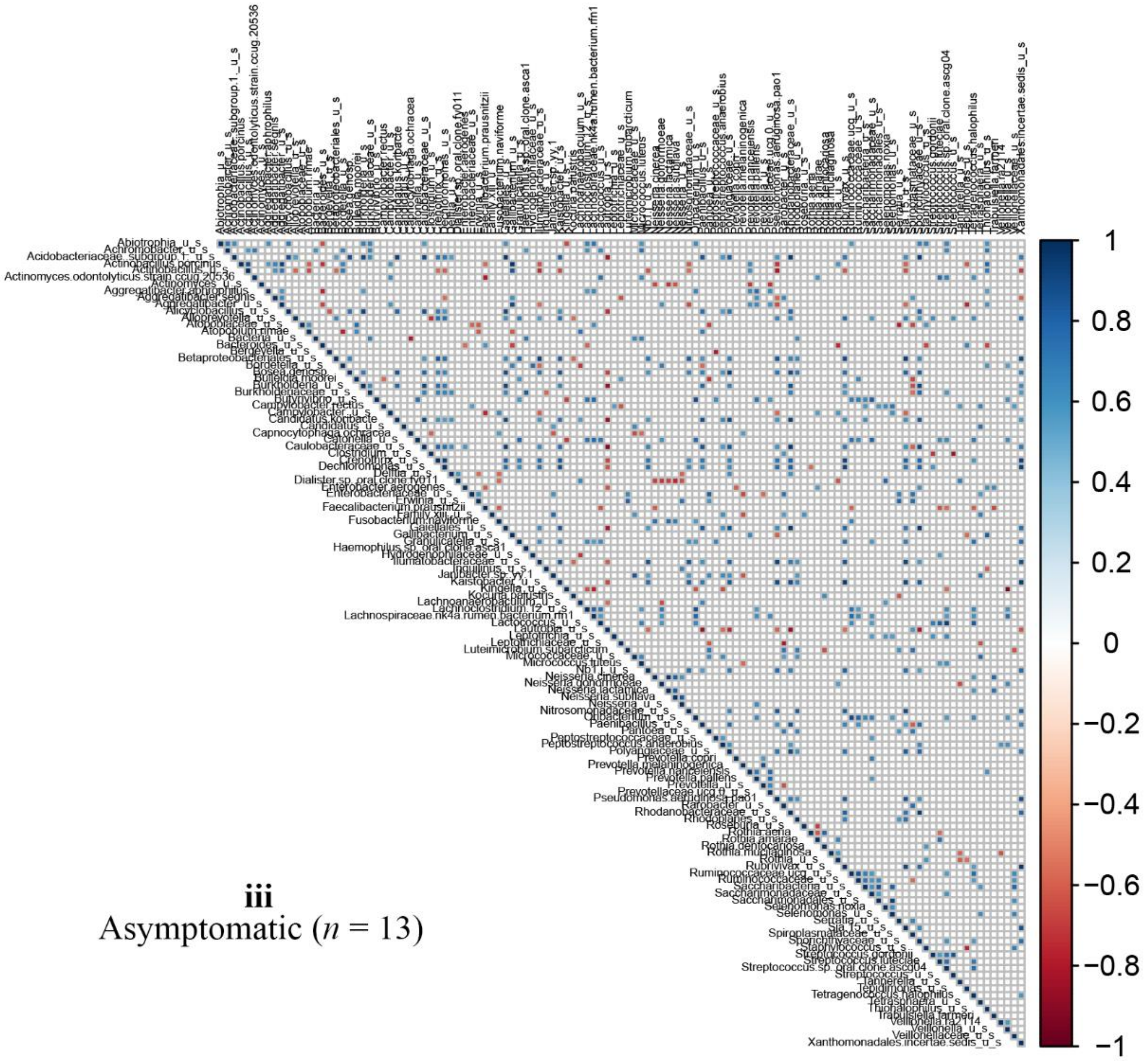

Supplementary Figure 3. Complete pairwise correlation matrix among all species in (i) healthy (*n* = 17), (ii) symptomatic (*n*=13) and (iii) asymptomatic (*n* = 13) populations showing shift in correlation patterns. Spearman correlation were generated using *corrplot* R package. Only significant correlation with $p<0.05$ were shown.

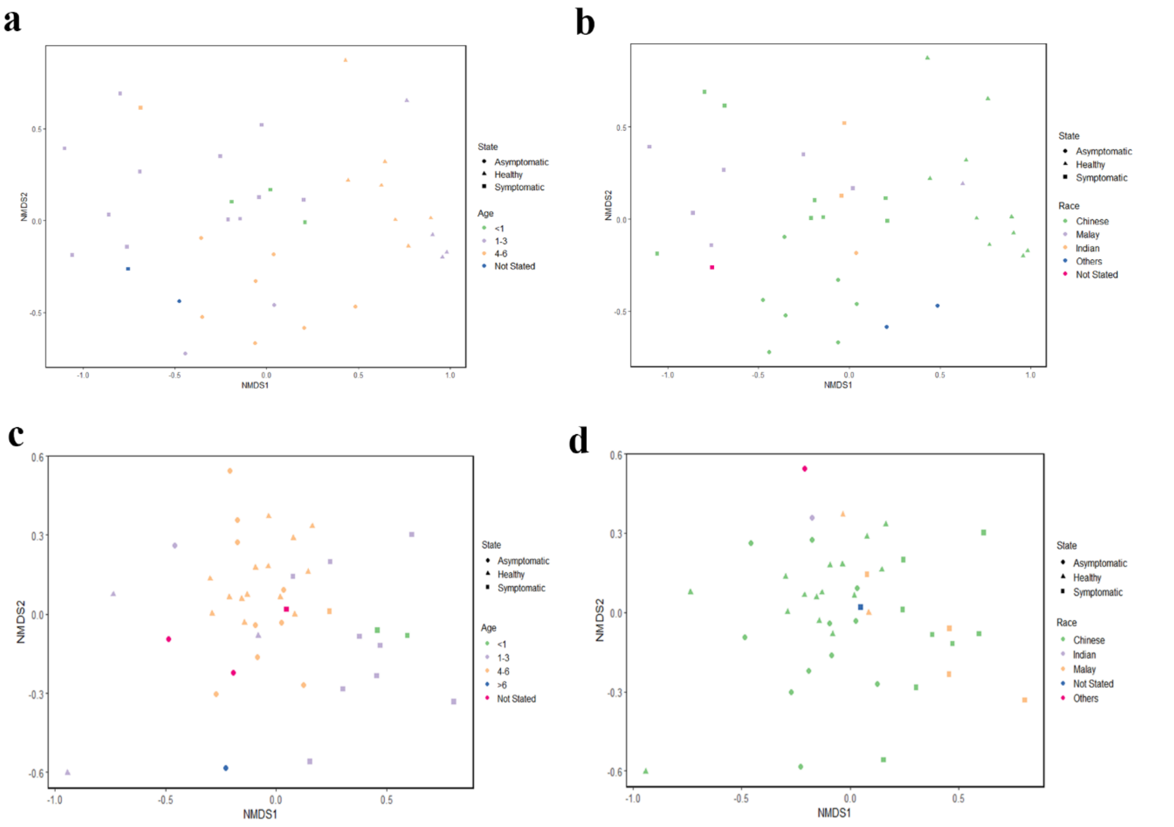

Supplementary Figure 4. Non-metric multi-dimensional scaling (NMDS) plot on virome with **(A)** age and **(B)** race as factors, and prokaryotic microbiome with **(C)** age and **(D)** race as factors.

Supplementary Table 1. Socio-demographic of healthy (*n* = 17) and asymptomatic (*n* = 13) cohort.

Socio-demographics	Asymptomatic (*n* = 17)	Healthy (*n* = 20)
	Frequency (%)	
**Age (Years)**		
2	0 (0.0)	2 (10.0)
3	2 (10.0)	2 (10.0)
4	4 (20.0)	6 (30.0)
5	4 (20.0)	7 (35.0)
6	4 (20.0)	3 (15.0)
7	1 (5.0)	0 (0.0)
Not stated	2 (10.0)	0 (0.0)
**Gender**		
Male	6 (30.0)	13 (65.0)
Female	11 (55.0)	7 (35.0)
**Race**		
Chinese	14 (70.0)	18 (90.0)
Malay	0 (0.0)	2 (10.0)
Indian	1 (5.0)	0 (0.0)
Others	2 (10.0)	0 (0.0)
**Previous HFMD history**		
TRUE	6 (30.0)	12 (60.0)
FALSE	11 (55.0)	8 (40.0)

Supplementary Table 2. Socio-demographic of symptomatic (*n* = 18) cohort.

**Symptomatic (*n* = 18)**	
**Socio-demographics**	**Frequency (%)**
**Age (Years)**	
≤1	10 (55.6)
2	5 (27.8)
3	1 (5.56)
4	1 (5.56)
Not Stated	1 (5.56)
**Race**	
Chinese	10 (55.6)
Malay	5 (27.8)
Indian	2 (11.1)
Not Stated	1 (5.56)
**Hospitalization duration**	
≤1 day	3 (16.7)
2 days	4 (22.2)
3 days	5 (27.8)
4 days	3 (16.7)
≥5 days	2 (11.1)
Not Stated	1 (5.56)
**Underlying conditions**	
None	11(61.1)
Eczema	2(11.1)
Blood Disorder	2(11.1)
Recurrent Tonsillitis	1(5.56)
Urinary Tract Infection	1 (5.56)
Not Stated	1 (5.56)

Supplementary Table 3. Contaminating viral sequences in metagenomic workflow identified using mock community with spiked in known viruses

**Mock community**		**Viruses detected in both mock community and clinical samples**
**Spiked in viruses**	**Viruses detected by metagenomic**	
Enterovirus A – CV-A6	Alphapapillomavirus 7	BeAn 58058 virus
Enterovirus A – CV-A16	Avian carcinoma virus	Bovine retrovirus CH15
Zika virus	Avian myelocytomatosis virus	Burkholderia phage KS10
Dengue virus 2	Baboon endogenous virus strain M7	Escherichia virus P1
Chikungunya virus	BeAn 58058 virus	Feline leukemia virus
	Bovine parvovirus 3	Getah virus
	Bovine retrovirus CH15	Human endogenous retrovirus K113
	Burkholderia phage KS10	Human immunodeficiency virus 1
	Dengue virus 4	
	Enterovirus B	Koala retrovirus
	Errantivirus	Onyong-nyong virus
	Feline leukemia virus	Reticuloendotheliosis virus
	Finkel-Biskis-Jinkins murine sarcoma virus	Semliki Forest virus
	Getah virus	Woodchuck hepatitis virus
	Gibbon ape leukemia virus	
	Hepatitis B virus	
	Human endogenous retrovirus K113	
	Human immunodeficiency virus 1	
	Invertebrate iridovirus 25	
	Jaagsiekte sheep retrovirus	
	Koala retrovirus	
	Murine leukemia virus	
	Mouse mammary tumor virus	
	Moloney murine leukemia virus	
	Onyong-nyong virus	
	Pestivirus H	
	RD114 retrovirus	
	Reticuloendotheliosis virus	
	Ross River virus	
	Semliki Forest virus	
	Ungulate erythroparvovirus 1	
	Vesicular stomatitis Indiana virus	
	West Nile virus	
	Woodchuck hepatitis virus	
	Y73 sarcoma virus	

Supplementary Table 4. NCBI BLAST results of sequences identified to be human mastadenovirus C. Only the top five hits from three representative sequences were shown.

Query ID	Top 5 hits from NCBI blast	Max Score	Total Score	Query Cover	E value	Per. ident	Accession
Human mastadenovirus C Representative sequence 1	Mutant Human adenovirus 2 isolate HAdV-C2-dE3B-CMV-GFP, complete sequence	244	244	100%	6.00E-61	100	MT277585.1
	Human mastadenovirus C isolate GD4163, partial genome	244	244	100%	6.00E-61	100	MN088492.1
	Human adenovirus 2 isolate SG06/HAdvC2/2016, complete genome	244	244	100%	6.00E-61	100	MN513342.1
	Human mastadenovirus C strain 44C2, partial genome	244	244	100%	6.00E-61	100	MH121111.1
	Human mastadenovirus C strain 42C2, partial genome	244	244	100%	6.00E-61	100	MH121109.1
Human mastadenovirus C Representative sequence 2	Human mastadenovirus C isolate Human/China/Shanghai/793/P2H2F2/2/2009, complete genome	322	322	100%	4.00E-84	100	MK836309.1
	Mutant Human adenovirus 2 isolate HAdV-C2-dE3B-CMV-GFP, complete sequence	322	322	100%	4.00E-84	100	MT277585.1
	Human mastadenovirus C isolate GD4163, partial genome	322	322	100%	4.00E-84	100	MN088492.1
	Human adenovirus 2 isolate SG06/HAdvC2/2016, complete genome	322	322	100%	4.00E-84	100	MN513342.1
	human adenovirus 2 isolate HK91, complete genome	322	322	100%	4.00E-84	100	MF044052.1
Human mastadenovirus C Representative sequence 3	Mutant Human adenovirus 2 isolate HAdV-C2-dE3B-CMV-GFP, complete sequence	623	623	100%	2.00E-174	100	MT277585.1
	Human mastadenovirus C strain 38C2, partial genome	623	623	100%	2.00E-174	100	MH121106.1
	Human mastadenovirus C strain 37C2, partial genome	623	623	100%	2.00E-174	100	MH121105.1
	Human mastadenovirus C strain 36C2, partial genome	623	623	100%	2.00E-174	100	MH121104.1
	Human mastadenovirus C strain 35C2, partial genome	623	623	100%	2.00E-174	100	MH121103.1

Supplementary Table 5. NCBI blast results for human herpesviruses detected in salivary virome. Only one representative sequence per species is shown.

Query Sequence ID	Top 5 hits from NCBI blast	Max Score	Total Score	Query C	E value	Per. Ident	Accession
Human betaherpesvirus 7 Representative Sequence	Human herpesvirus 7 strain RK, complete genome	12067	1.08E+05	99%	0	98.98%	AF037218.1
	Human herpesvirus 7 isolate UCL-1, partial genome	12061	1.04E+05	99%	0	98.97%	KF558370.1
	Human herpesvirus-7 (HHV7) JI, complete virion genome	12050	1.08E+05	99%	0	98.94%	U43400.1
	Human herpesvirus 7 genes for major capsid protein and capsid protein, complete cds	1378	4364	4%	0	99.47%	D32005.1
	Human herpesvirus 7 glycoprotein gp65 (gp65) mRNA, complete cds	1081	2346	2%	0	97.19%	AF198085.1
Human betaherpesvirus 5 Representative Sequence	Human betaherpesvirus 5 strain SYD-SCT2, complete genome	470	3822	100%	2.00E-127	99.61%	MT044480.1
	Human betaherpesvirus 5 strain HAN-SOT5, complete genome	470	3833	100%	2.00E-127	99.61%	MT044479.1
	Human betaherpesvirus 5 strain HANSCTR13, complete genome	470	3780	100%	2.00E-127	99.61%	KY490088.1
	Human betaherpesvirus 5 strain NL/Rot3/Nasal/2012, partial genome	470	3794	99%	2.00E-127	99.61%	KT726942.2
	Human betaherpesvirus 5 strain NL/Rot2/Urine/2012, partial genome	470	3868	99%	2.00E-127	99.61%	KT726941.2
Human betaherpesvirus 6B Representative Sequence	Human betaherpesvirus 6B isolate iciHG02016, partial genome	1463	38906	82%	0	100.00%	MG894372.1
	Human betaherpesvirus 6 strain 02-543-S1a, partial genome	1458	60389	99%	0	99.87%	MF511175.2
	Human betaherpesvirus 6 strain NY-405, partial genome	1458	44074	83%	0	99.87%	KY290219.2
	Human betaherpesvirus 6 strain NY-393, partial genome	1458	43671	82%	0	99.87%	KY290215.2
	Human betaherpesvirus 6 strain NY-353, partial genome	1458	43265	82%	0	99.87%	KY290209.2
Human gammaherpesvirus 4 Representative Sequence	Human gammaherpesvirus 4 strain rMSHJ, complete genome	5921	2.11E+05	99%	0	98.74%	MK973062.1
	Human gammaherpesvirus 4 DNA, nearly complete genome, strain: HNNPC4	5893	2.15E+05	99%	0	98.59%	LC150337.1
	Human gammaherpesvirus 4 DNA, nearly complete genome, strain: HNNPC3	5893	2.14E+05	99%	0	98.59%	LC150327.1
	Human gammaherpesvirus 4 DNA, nearly complete genome, strain: HNNPC6	5888	2.09E+05	99%	0	98.57%	LC150741.1
	Human gammaherpesvirus 4 sLCL-T12.18 DNA, complete genome	5888	2.28E+05	98%	0	98.57%	LC573552.1

Supplementary Table 6. NCBI blast results of representative human herpesviruses sequences detected in salivary virome with search limited to *homo sapiens*.

Query Sequence ID	Description	Max Score	Total Score	Query Cover	E value	Per. Ident	Accession
Human betaherpesvirus 7 Representative Sequence	Homo sapiens isolate preeclampsia-29 endogenous virus endogenous human herpesvirus 6, complete sequence	182	305	1%	5.00E-41	76.44%	MT508968.1
Human betaherpesvirus 5 Representative Sequence	Homo sapiens neurite extension and migration factor (NEXMIF), mRNA	56.5	56.5	1%	1.00E-04	100.00%	NM_001008537.3
	Homo sapiens neurite extension and migration factor (NEXMIF), RefSeqGene on chromosome X	56.5	56.5	1%	1.00E-04	100.00%	NG_027726.1
	Homo sapiens cDNA, FLJ97419	56.5	56.5	1%	1.00E-04	100.00%	AK307471.1
	Human DNA sequence from clone RP13-9D14 on chromosome X, complete sequence	56.5	56.5	1%	1.00E-04	100.00%	AL390035.10
Human betaherpesvirus 6B Representative Sequence	Homo sapiens isolate preeclampsia-31 endogenous virus endogenous human herpesvirus 6, complete sequence	1452	60766	100%	0	99.75%	MT508970.1
	Homo sapiens isolate preeclampsia-30 endogenous virus endogenous human herpesvirus 6, complete sequence	1452	60764	100%	0	99.75%	MT508969.1
	Homo sapiens isolate preeclampsia-28 endogenous virus endogenous human herpesvirus 6, complete sequence	1452	60887	99%	0	99.75%	MT508967.1
	Homo sapiens isolate preeclampsia-27 endogenous virus endogenous human herpesvirus 6, complete sequence	1452	60764	100%	0	99.75%	MT508966.1
	Homo sapiens isolate preeclampsia-25 endogenous virus endogenous human herpesvirus 6, complete sequence	1452	60943	100%	0	99.75%	MT508965.1
Human gammaherpesvirus 4 Representative Sequence	Human DNA sequence from clone DASS-285K8 on chromosome 6, complete sequence	1190	119000%	6%	0.00%	99.24%	BX248579.4
	Homo sapiens clone SKT05-D4 putative promoter sequence	459	45900%	2%	5.00E-125	99.60%	AY270836.1
	Homo sapiens clone SKT01-F10 putative promoter sequence	448	44800%	2%	1.00E-121	98.80%	AY270254.1
	Homo sapiens clone SKT07-E11 putative promoter sequence	407	407	2%	2.00E-109	98.69%	AY270927.1
	Homo sapiens clone SKG03-G02 putative promoter sequence	401	401	2%	8.00E-108	98.25%	AY270599.1

Supplementary Discussion

We recognized that contamination is inevitable in metagenomics and have employed various strategies pre- and post-analysis to ensure the reliability of our results. One of the strategies is the use of mock community with known viruses to determine the background contaminating viruses from environment and reagents. Due to our experimental setup involving pooling of individual libraries and subjecting them to enrichment workflow, mock community was not directly spiked in the actual sequencing runs to avoid overrepresentation and masking by a single library during sequencing. As such to avoid masking, mock communities were sequenced every 3 runs throughout the study using the same workflow as the saliva samples. Any viral reads that were not part of the spiked in purified viruses in the mock community were considered as contaminant and removed.

With a high detection rate of viruses such as human mastadenovirus C and human herpesvirus, we were concerned if these viruses that were found across almost all samples were true positive. Given that these viruses have highly repetitive region, de novo assembly could be challenging due to similarity to other genomes which could lead to false identification. To tackle this, BLAST analysis was conducted in post for sequences identified to be human mastadenovirus C and human herpesviruses to confirm the alignment of each virus **(Supplementary Table 4 and Supplementary Table 5).** Sequences were also checked for possibility of originating from human genomes by limited the BLAST search parameters to *Homo sapiens*. Of the 4 types of herpesviruses detected in this study, human betaherpesvirus 6b matches endogenous herpesvirus 6b at 99.75% **(Supplementary Table 6)**. Human betaherepesvirus 6b is known to be integrated in the human chromosome[1], as such, it is likely that it was derived from the human genome and thus was excluded from downstream analysis. Similarly, human gammaherpesvirus 4 shows an identity of 99.24% to the human genome on chromosome 6 and was also excluded from the downstream analysis **(Supplementary Table 6)**. However, sequences identified as human betaherpesvirus 7 do not show significant matches to any endogenous human herpesviruses except for a conserved region of endogenous human herpesvirus 6 for up to 700 nucleotides out of 42, 443 nucleotides (~1%). **(Supplementary Table 6).** In a similar fashion, detected human betaherpesvirus 5 sequences matches to the human genome at about a small region of 30 nucleotides out of 1984 nucleotides or with no significant matches. This suggests that it is unlikely that human betaherpevirus 5 and 7 were derived from the human genome. While human mastadenovirus C was identified to be a reagent contaminant by other study[2], it is linked to QIAamp DNA kit which was not used in our workflow. The absence of human mastadenovirus C sequences from our mock community further confirms that it is unlikely to be a reagent contaminant.

While CV-A5 genomes detected are very closely related as compared to other species such as CV-A6, they were shown to be not identical when pairwise distances were computed between the CV-A5 sequences. As CV-A6 infection is a lot more common and severely endemic in Singapore, allowing higher mutation events and genetic diversity as compared to CV-A5. Further surveillance study on the asymptomatic population is needed to identify the true prevalence of CV-A5.

Apart from enterovirus, other enteric viruses such as rotavirus was also detected. Notably, all three samples which harbored rotaviruses were symptomatic samples that were collected from hospitals. Although there is a possibility of nosocomial transmission from the hospital environment as they were reported to have long environmental persistence[3], replication of rotaviruses in the human oral cavity is currently not known or well-studied. Interestingly, other enteric virus, human enterovirus 71, was found to be able to replicate in the tonsil[4], and as such, a large scale surveillance study could be done to confirm the possibility of extra-enteric organ involvement in the rotavirus infection. On the other hand, there is also a slight change that the virus detected in the oral cavity without any replication at the site due to inoculation from the environment.

In this study, V3-V4 regions of 16S rRNA hypervariable regions were targeted for the identification of bacterial species in the saliva samples. However, the use of V3-V4 regions of 16S rRNA gene could potentially underestimate species diversity as there is a tendency of merging several species into a single OTU.[5] Having this in mind, we recognized that there could be insufficient species-level resolution in certain instances and therefore diversity may

be underestimated. As such, we reported that the number of species detected per genera is likely to be the minimum number. Species correlation was reported when species-level typing was possible, in cases when only genus was reported, the species is unknown.

Supplementary References

1. Telford, M., Navarro, A. & Santpere, G. Whole genome diversity of inherited chromosomally integrated HHV-6 derived from healthy individuals of diverse geographic origin. Scientific Reports 8, (2018).
2. Asplund, M. et al. Contaminating viral sequences in high-throughput sequencing viromics: a linkage study of 700 sequencing libraries. Clinical Microbiology and Infection 25, 1277-1285 (2019).
3. Boone, S. & Gerba, C. Significance of Fomites in the Spread of Respiratory and Enteric Viral Disease. Applied and Environmental Microbiology 73, 1687-1696 (2007).
4. Xie, G. et al. Susceptibility of human tonsillar epithelial cells to enterovirus 71 with normal cytokine response. Virology 494, 108-118 (2016).
5. Bukin, Y. et al. The effect of 16S rRNA region choice on bacterial community metabarcoding results. Scientific Data 6, (2019).
