# Supplementary material for: Characterization of oral virome and microbiome revealed distinctive microbiome disruptions in paediatric patients with hand, foot and mouth disease
Source: NPJ Biofilms Microbiomes. 2021 Feb 19;7:19. doi: 10.1038/s41522-021-00190-y (PMC7895916; doi:10.1038/s41522-021-00190-y)
Supplement: Supplementary file 2 — Reporting Summary [file 41522_2021_190_MOESM2_ESM.pdf]

nature research

Corresponding author(s): Justin Jang Hann Chu

Last updated by author(s): 14/1/2021

Reporting Summary

Nature Research wishes to improve the reproducibility of the work that we publish. This form provides structure for consistency and transparency in reporting. For further information on Nature Research policies, see our Editorial Policies and the Editorial Policy Checklist.

Statistics

For all statistical analyses, confirm that the following items are present in the figure legend, table legend, main text, or Methods section.

n/a	Confirmed	
☐	☒	The exact sample size (*n*) for each experimental group/condition, given as a discrete number and unit of measurement
☐	☒	A statement on whether measurements were taken from distinct samples or whether the same sample was measured repeatedly
☐	☒	The statistical test(s) used AND whether they are one- or two-sided<br>*Only common tests should be described solely by name; describe more complex techniques in the Methods section.*
☐	☒	A description of all covariates tested
☐	☒	A description of any assumptions or corrections, such as tests of normality and adjustment for multiple comparisons
☐	☒	A full description of the statistical parameters including central tendency (e.g. means) or other basic estimates (e.g. regression coefficient) AND variation (e.g. standard deviation) or associated estimates of uncertainty (e.g. confidence intervals)
☐	☒	For null hypothesis testing, the test statistic (e.g. *F*, *t*, *r*) with confidence intervals, effect sizes, degrees of freedom and *P* value noted<br>*Give P values as exact values whenever suitable.*
☒	☐	For Bayesian analysis, information on the choice of priors and Markov chain Monte Carlo settings
☒	☐	For hierarchical and complex designs, identification of the appropriate level for tests and full reporting of outcomes
☒	☐	Estimates of effect sizes (e.g. Cohen's *d*, Pearson's *r*), indicating how they were calculated

Our web collection on statistics for biologists contains articles on many of the points above.

Software and code

Policy information about availability of computer code

Data collection	The virome sequencing data were generated via the Illumina HiSeq sequencing platform and 16S rRNA sequencing data were generated via the Illumina Novaseq6000 sequencing platform.
Data analysis	The following were used for data analysis:<br>1. Genome Detective version 1.126<br>2. R version 1.2.1335<br>3. Jamovi software package version 0.9.6.9<br>4. Geneious Prime 2020.1.2<br>5. CosmosID 1.0.0<br>6. ggplot2 package version 3.3.2<br>7. corrplot package version 0.84<br>8. vegan package version 2.5-6<br>9. Performance Analytics package version 2.0.4<br>10. Clustvis (BETA)

For manuscripts utilizing custom algorithms or software that are central to the research but not yet described in published literature, software must be made available to editors and reviewers. We strongly encourage code deposition in a community repository (e.g. GitHub). See the Nature Research guidelines for submitting code & software for further information.

Data

Policy information about availability of data

All manuscripts must include a data availability statement. This statement should provide the following information, where applicable:

- Accession codes, unique identifiers, or web links for publicly available datasets
- A list of figures that have associated raw data
- A description of any restrictions on data availability

Data availability statement is included in the manuscript.

Field-specific reporting

Please select the one below that is the best fit for your research. If you are not sure, read the appropriate sections before making your selection.

☒ Life sciences ☐ Behavioural & social sciences ☐ Ecological, evolutionary & environmental sciences

For a reference copy of the document with all sections, see nature.com/documents/nr-reporting-summary-flat.pdf

Life sciences study design

All studies must disclose on these points even when the disclosure is negative.

Sample size	Sample size was not determined in prior. Possible effect size was not being able to be pre-determined due to the novelty of the study and the lack of previously deposited oral virome data in repositories. To counteract this for a reasonable statistical power, the deep sequencing method with an average reads of 40 million per sample for virome and 146, 978 reads per sample for prokaryotic microbiome were used respectively in this study.
Data exclusions	Data were not excluded from analysis
Replication	N/A
Randomization	Samples were chosen randomly for each cohort.
Blinding	Investigators were not blinded during experiment as it is not relevant in this study.

Reporting for specific materials, systems and methods

We require information from authors about some types of materials, experimental systems and methods used in many studies. Here, indicate whether each material, system or method listed is relevant to your study. If you are not sure if a list item applies to your research, read the appropriate section before selecting a response.

Materials & experimental systems

n/a	Involved in the study	
☒	☐	Antibodies
☒	☐	Eukaryotic cell lines
☒	☐	Palaeontology and archaeology
☒	☐	Animals and other organisms
☐	☒	Human research participants
☒	☐	Clinical data
☒	☐	Dual use research of concern

Methods

n/a	Involved in the study	
☒	☐	ChIP-seq
☒	☐	Flow cytometry
☒	☐	MRI-based neuroimaging

Human research participants

Policy information about studies involving human research participants

Population characteristics	The details of the social demographics of the participants is included in supplementary table 1 and 2.
Recruitment	Paediatrics patients admitted for symptomatic HFMD diagnosed by clinicians were enrolled from period of 2013 to 2018 from KK Women's ad Children's Hospital. Healthy volunteers were routinely collected from childcare centers across Singapore. Volunteers were screened for absence of fever, rashes, blisters and oral ulcers during sample collection. No potential self-selection bias or other biases is present during participants recruitment.
Ethics oversight	Centralized Institutional Review Board (CIRB) of Singhealth, National University of Singapore Institutional Review Board (NUS-

Ethics oversight	IRB)

Note that full information on the approval of the study protocol must also be provided in the manuscript.
